# “Nothing Is Gonna Change If We Don't Care for Everyone”: A Narrative Inquiry Alongside Urban Indigenous Youth in an Afterschool Physical Activity Wellness Program

**DOI:** 10.3389/fspor.2022.822547

**Published:** 2022-09-14

**Authors:** Brian Lewis, Lee Schaefer, Sean Lessard, Jordan Koch

**Affiliations:** ^1^Education, University of Regina, Regina, SK, Canada; ^2^College of Kinesiology, University of Saskatchewan, Saskatoon, SK, Canada; ^3^Secondary Education, University of Alberta, Edmonton, AB, Canada; ^4^Department Kinesiology and Physical Education, McGill University, Montreal, QC, Canada

**Keywords:** Indigenous youth, after-school program, peer-mentorship, narrative inquiry, youth voice

## Abstract

In the fall of 2013, the authors received funding to help develop and implement an afterschool wellness program alongside Indigenous youth aged 6–10 years old in the North Central neighborhood of Regina, Saskatchewan, Canada. The Growing Young Movers (GYM) afterschool program was funded, in part, as a corrective response to a broader social trend in which Indigenous youth in this neighborhood reported declining health and wellness outcomes, as well as multiple other barriers to social inclusion. This article discusses the reflections of three senior high school Indigenous youth (16–18 years old) who participated in the afterschool program as peer-mentors over a 2-year period from 2015 to 2017. Our inquiry reveals how these youth viewed the program—and their role(s) within it—in far more complex, active, and even political terms, than the program's initial framing as a physical activity-based “intervention” had anticipated. Our analysis (re)positions youth according to their own personalized voice and narratives as: cultural leaders, knowledge holders, and as agents of change in their community.

## Introduction

Education scholars have drawn important attention to the merits of a “wrap-around” approach in afterschool youth programs to balance schoolwork, career training, and employment opportunities, with various other social activities, such as sports and other extracurricular activities (Kugler, 2001; Fashola, 2002; Lauer et al., 2006; Kremer et al., 2015; Gaudreault et al., 2016). Education scholars have also prioritized a results-orientated approach when evaluating the impacts of afterschool programming, privileging markers such as crime and gang prevention, reduced substance use and abuse, and various academic outputs over other qualitative outcomes (Taheri and Welsh, 2016; Budd et al., 2020). Considerably less scholarly attention has been devoted to understanding the meanings that youth themselves derive from such programming (Hopper and McHugh, 2020)—an oversight that risks limiting our analyses to only a select few behavioral outcomes that afford little insight into why youth join such programs in the first place and stay involved for long periods of time.

In the fall of 2013,^1^ the authors received funding to help design and implement an afterschool program for urban Indigenous youth in the North Central neighborhood of Regina, the capital city of the western Canadian province of Saskatchewan. The Growing Young Movers (GYM) afterschool program was funded as a partial response to a broader social trend in which Indigenous youth in North Central Regina reported multiple barriers to social inclusion, as well as poorer health and wellness outcomes. The official purpose of the weekly afterschool program was to provide a cost-free and consistently delivered holistic wellness and movement experience for urban Indigenous youth aged 6-to-10 years old. In addition, the afterschool program was also viewed as an opportunity for high school Indigenous youth aged 16-to-18 to gain relevant employment experience as mentors for the younger youth.

This paper discusses the experiences of three high school Indigenous youth who all served as peer-mentors within the afterschool program over a 2-year period from 2015 to 2017. Adopting narrative inquiry as our primary methodology, our study revealed that the three Indigenous peer-mentors defined their experiences according to a series of deliberate and largely empowering agendas in which they envisioned themselves as cultural leaders, knowledge holders, and as active agents of change within their community. These findings stand in contrast to dominant narratives that often persist within popular and some scholarly accounts of afterschool programming in which youth participants are cast as passive consumers of a program's official messaging (Donnelly and Coakley, 2002; Coalter, 2010; Coakley, 2011)—messaging that often prioritizes discipline over creativity, and individual self-control over youth-engineered, self-determined, social change. In what follows, we draw from the reflections of three peer-mentors who described themselves as actively engaged in a struggle for personal, cultural, and community (re)construction against a backdrop of structural violence and urban marginality.

## The Backdrop: North Central Regina

The City of Regina—pop. 241,442—is in the southern third of the province of Saskatchewan, along the northern borders of the American states of Montana and North Dakota. Located in Treaty 4 territory, Regina is the original territory of the Cree, Ojibwe, Saulteaux, Dakota, Nakota, Lakota, as well as the homeland of the Métis Nation and is home to one of the largest urban Indigenous populations in Canada. Approximately 10% of Regina's total population self-identify as Indigenous (double the national percentage of 5%), with Indigenous youth under 20 years old representing nearly half of Regina's Indigenous population (Statistics Canada, 2013; Indigenous Saskatchewan Encyclopedia, 2021).

Historically, Regina's economy has been driven by various agricultural activities, as well as the development of numerous other natural resources, most notably oil, natural gas, and potash. Significant growth in these sectors over the early 2000s, especially in natural resource production, transformed Saskatchewan into a *have* province. Statistics Canada (2016) placed Regina's unemployment rate at 5%—a number that has increased significantly in recent years following the collapse of global oil prices in 2014, as well as numerous other hits to the potash and natural gas industries. According to Economic Development Regina, the unemployment rates in Regina are projected to reach 6.4% in 2021, and are likely even higher now given the trends with the COVID pandemic.

Conversely, even as the province boomed in the early 2000s, the unemployment rate for Regina's Indigenous population remained at a strikingly high 12.1% in 2008 (Statistics Canada, 2011), and has continued to increase to 14.2% according to the 2016 National Household survey. Significant gaps within education, health services, the criminal justice system, and child welfare have further entrenched the systemic inequalities between Indigenous and non-Indigenous populations in Saskatchewan. For example, the high school graduation rate for Indigenous youth was almost half (43.2%) that of the province's non-Indigenous youth (85.4%) in 2018 (Saskatchewan Ministry of Education, 2009). The presence of Indigenous peoples within Saskatchewan's criminal justice system was also significantly inflated in 2018 compared to the province's non-Indigenous population, with Indigenous peoples representing 74% of adult admissions into custody despite comprising only 16% of the province's total population (Clermont et al., 2019).

Such racialized inequalities underscore the complex legacy and ongoing manifestations of settler-colonialism in Canada—and in Saskatchewan specifically—and the categorical failure of successive levels of Canadian government to reconcile the long-standing social issues associated with this history (Miller, 1993, 2003; Lavallee and Poole, 2010; Daschuk et al., 2013; Razack, 2014). Recent months have further highlighted these racialized discrepancies as policies linked to the COVID-19 virus have severely limited the retail and service industries in which a disproportionately high number of Indigenous and other racialized minorities are employed (Arriagada et al., 2020). Many urban Indigenous, First Nations, and Métis communities have also been among the hardest hit by COVID-19 throughout the province, as overcrowded homes and underfunded social and healthcare services have fueled the spread of the virus (Vescera, 2020).

The North Central community of Regina has been hit especially hard by these collective forces. Canadian Journalist Gatehouse's (2017) analysis of “what's wrong” in North Central found that the community remains a site of controversy and neglect: “The neighborhood has been the scene of 10 of the city's 17 murders over the past 2 years. Gun crime is up substantially, so is methamphetamine use. Overall, this is an area that is home to 4.5% of Regina's population and continues to account for 17% of its crime” (Gatehouse, 2017). However, despite the habitual framing of North Central as violent and crime ridden, our research discovered a host of counternarratives that also predominate the area, especially among youth. The lead author (Brian) was working as a physical educator in North Central at the time that funding for this project was awarded. Brian was confronted daily by the area's limited youth programming, as well as the striking leadership potential of North Central youth. In what follows, we outline the collaborative process that led to the creation of a GYM program at a North Central elementary school as part of a broader research investigation into the everyday lives and aspirations of local youth from the area.

## Methodology

Researchers have drawn important attention to the historically tenuous relationship that exists between university researchers and Indigenous communities in Canada (Ermine, 1995; Battiste, 2008; Kovach, 2009; Henry and Tait, 2017). As the Gixtaala anthropologist Charles Menzies (2001, p. 22) explained, “Underlying the contemporary relations between researchers and Indigenous peoples is a history of forced relocation, systematic discrimination, and expropriation of resources and territory.” We sought to mitigate this tenuous history by building relationships as part of the ontological commitments of narrative inquiry research and relational ethics (Clandinin et al., 2018). The research team met regularly with a wide range of community members in North Central, including Indigenous youth, family members, Elders, schoolteachers, and administrators, as well as members of community school councils and community agencies that had long-standing relations with the community. These early conversations generated a youth driven and community-guided framework for the afterschool program that was pursued throughout our research, as well as nurtured an ethical research space (Ermine, 2007) that promoted ongoing relationships among various community members, i.e., “the foundational components to Indigenous research” (Henry and Tait, 2017, p. 183).

An important part of the community-guided framework was that our research prioritized the voices and experiences of Indigenous youth within the afterschool program. To that end, we employed narrative inquiry as our primary methodology to understand the lived experiences of three senior high school students participating as mentors in the afterschool program (Clandinin and Connelly, 2000). Narrative inquiry is rooted in the early works of Dewey (1938, 1958)—a classical pragmatist best known for positioning pragmatism alongside educational reform. Like Dewey (1938), narrative inquirers view human experience as accumulative knowledge for living. More precisely, narrative inquirers view people as knowledge holders and center peoples' experiences within all phases of the research process (Clandinin, 2013).

In the context of schooling, Connelly and Clandinin (2006) referred to teachers' identities as stories to live by: “Stories to live by are shaped by such matters as secret teachers' stories, sacred stories of school, and teachers' cover stories” (p. 4). A person's “stories to live by” are shaped by cultural, social, familial, and institutional narratives, but also shape the landscapes within which they live (Clandinin and Connelly, 1995). While Clandinin and Connelly conceptualized the school as a professional knowledge landscape, we conceptualized the afterschool program as a community landscape where youth live, play, work, and grow together with help from others. Narrative conceptions further position youth as knowledge holders who, like teachers and other community members, shape and (re)construct their landscape according to their own values and experiential practices (Clandinin et al., 2014).

In terms of the afterschool program, community members were clear from the outset that youth needed opportunities to play and to be physically active, and to socialize in a safe, supervised, and positive social environment. Both physical education and wellness (broadly conceived) provided the core content areas around which the afterschool program was structured. Community Elders also voiced the need for programming to enhance students' cultural wellbeing through purposeful movement opportunities connected to traditional land-based activities. The feedback from this collaborative process further resulted in an inter-generational approach to mentorship within the program—an approach that engaged Indigenous children (primary elementary students), Indigenous youth (middle and high school students), volunteers (university pre-service teachers), as well as the researchers, in various socio-cultural and physical activity experiences within the gymnasium and broader community.

Each Wednesday for ~2-h, at the end of the school day, a group of 20–25 elementary-aged Indigenous youth and 6 senior high school peer-mentors met at the school gymnasium to engage in physical activity led by Brian (the first author). Brian co-facilitated these weekly sessions alongside the youth mentors, who also helped to organize snacks, group outings and discussions, and various other social and cultural activities. While the school gymnasium provided the primary meeting spot for our weekly activities, the group also traveled to various other locations throughout the city (e.g., museums, tobogganing hills, the university, a gymnastics club, etc.). Some of our more common activities included developmental games and physical activities that were modified to optimize student engagement across all ages and skill levels. The group also regularly engaged in guided conversations led by the peer mentors that prioritized youth voices to ensure that all activities remained inclusive, and youth driven.

In terms of qualitative data, the research team accumulated a variety of field texts over the 4-year period, including observational data, conversational data, digital stories, and program evaluation surveys. In this paper, we narratively inquire into the conversational data to shed light on the experiences of three Indigenous youth mentors from the afterschool program: Candice, Clary, and Colin^2^. Colin had attended the elementary school at which the program was offered, and had expressed desire to volunteer with the program since grade 7. Clary was looking for meaningful volunteer work to help bolster her post-secondary application, and thus became involved as a peer-mentor in the program. Candace had a younger sibling who participated in the program whom she regularly picked her up after school. Candace involved herself in the program to connect with her sister and to help pass the time. All three peer-mentors consented to converse with the research team outside of the program as part of this narrative inquiry.

In total, Brian engaged in five formal research conversations with each research participant, asking questions that explored their personal experiences with the program, as well as their hopes and dreams for the community. Following Clandinin and Connelly (2000), special attention was paid to the *social* (what was happening around them), *temporality* (how their experiences were bound in time), and finally, *place*, “which attends to the specific concrete physical and topological boundaries of inquiry landscapes” (p. 51). Brian framed the research puzzle with “a sense of a search, a ‘re-search,' a searching again… a sense of continual reformulation” (Clandinin and Connelly, 2000, p. 124). This promoted an openness to our conversations that allowed both “participants and researchers opportunities to further co-compose storied interpretations and to negotiate the multiplicity of possible meanings” (Clandinin, 2013, p. 47). Alongside each participant, Brian mapped out their respective narrative accounts with constant feedback and input from Candice, Clary, and Colin. The research team next explored all three narrative accounts for common threads or, as Clandinin and Connelly (2000, p. 132) described, for “resonances or echoes that reverberated across accounts.” The resonances identified included themes such as play, school and education, community, as well as cultural narratives that animated youths' experiences within the afterschool program.

## Theoretical Frame—Live, Tell, Retell, and Relive

Clandinin (2013) explained that we all *live, tell, retell*, and *relive* our experiences through stories. Each phase of *living, telling, retelling*, and *reliving*, contains meanings in narrative inquiry that helped to create a framework for this paper. Prior to Candice, Clary, and Colin joining the afterschool program they were indeed living and telling stories. As human beings, we all *live*, and then *tell* stories about this living (Clandinin, 2013). Brian's lived and told stories also began to interact with the stories of Candace, Clary, and Colin within the afterschool program. This is part of what Clandinin (2013) described as a relational interaction—a process that was crucial to help mitigate various critiques of interpretative bias (Blumenreich, 2004; Nunkoosing, 2005; Leskelä-Kärki, 2008; Gard, 2014). Brian and the youths came to know each other through their weekly interactions, learning each other's stories, and, in turn, building a relationship that fostered even greater depth to our narrative inquiry.

Drawing from Dewey's (1938) theorization of experience, Clandinin and Connelly (2000) developed a metaphorical three-dimensional narrative inquiry space that attends to the three commonplaces of place, temporality, and sociality. The commonplace of temporality is influenced by Dewey's view of experience with respect to continuity and interaction. For Clandinin and Rosiek (2007, p. 41) inquiry exists “within a stream of experience that generates new relations that then becomes a part of future experience.” In thinking this way, narrative inquirers envision an interconnectedness between past experiences and the stories people tell and retell about their lives and past experiences. Such stories “are the result of a confluence of social influences on a person's inner life, social influences on their environment, and their unique personal history” (Clandinin and Rosiek, 2007, p. 41). As Clandinin (2013, p. 34) further explained, “as we retell or inquire into stories, we may begin to shift the institutional, social, and cultural narratives in which we are embedded” (p. 34). Brian observed such shifts in terms of how the youth viewed themselves—and their role(s) within the program—in far more complex, active, and even political terms, than the program's initial framing as a physical activity-based ‘intervention' had anticipated. In what follows, we position youth according to their own narratives as: cultural leaders, knowledge holders, and as agents of change in their community.

### Knowledge Landscapes

Candice, Clary, and Colin were *living* and *telling* stories related to multiple landscapes long before their involvement in the afterschool program. Collectively, we theorized the afterschool program as another knowledge landscape upon which they lived and imprinted their stories. Our inquiry focused on the affinities, differences, and complexities between the various community knowledge landscapes in which the youth lived and (re)constructed their identities. Following Clandinin (2013, p. 40), we wanted to explore the “cultural, social, institutional, familial, and linguistic narratives” that shaped youths' stories with respect to the afterschool program and the broader community of Regina. What stories were Candice, Clary, and Colin living and telling in the GYM program? What stories did they bring with them to the program? Finally, how did these stories shape their strategies for living or, as Connelly and Clandinin (1999) described, their *stories to live by*?

## Results

### The Grand Narrative

Greatness ***isn't always easy***.
Do not ***doubt*** yourself.
***Anyone can be successful*** so long as you want to be.
(Field Text, January 2014)

These three lines are part of a short piece that Clary wrote as an introduction to be read to the children in the afterschool program a few months after she herself had joined. Clary and Brian revisited these words whilst reviewing her narrative account one final time. Curiously, Clary elected to rearrange the words to reflect a popular stereotype of Indigenous youths in North Central Regina as hopeless and flawed:

Greatness ***isn't*** *always easy* ***possible.***

Do not ***Doubt*** yourself.

***Anyone can be successful*** so long as you want to be are not Indigenous.

Clary's edited version of the original text revealed her frustration with how Indigenous youths in North Central have been stereotyped. Colin expressed similar frustrations with how Indigenous youths have been treated in the area: “…sometimes when I'm in a store or something, there'd be people following me around. I don't really like it very much” (3, p. 19)^3^. All three mentors recalled experiences of profiling and marginalization within and beyond North Central, which contributed to feelings of frustration, anger, and loneliness. Colin also recognized the degenerative affect that experiences such as these had on his own mental health and wellbeing: “When I am getting followed around, for some reason, I feel guilty or it's weird. I don't really feel good. It's not good for my wellbeing, I guess” (3, p. 19).

Candice grew up hearing these narratives and referred to racial profiling as misguided assumptions that unfairly position Indigenous youth as risks to both themselves and to the broader public: “Many people assume what First Nation people are... they didn't finish school, they don't have jobs, they're living off welfare and they have lots of kids and maybe alcohol and drugs” (3, p. 19). She sensed the intrusive and judgmental gaze of non-Indigenous neighbors each time the GYM program ventured beyond the North Central community:

When we go out, when we take the kids on outings, it's like mostly when people see us, it's like a bunch of First Nations kids and First Nations mentors, and they assume that it's going to be hard with us there, or they're going to have troubles with us.

(3, p. 8)

For Brian, the racialized stereotypes confronted by Candice, Clary, Colin, contrasted markedly with the roles and identities exhibited within the afterschool program. Brian knew Colin as a leader—a person he could depend on and to whom the kids looked up and literally clung to. Candice, likewise, exhibited so much compassion for her younger siblings and proved to be a natural mentor for many of the other kids in the GYM program. Yet, it was clear that Colin, Clary, and Candice were negatively impacted by the racism and stereotypes they encountered on a daily basis. As Clary explained,

Would you think that I was going to be successful in high school? Probably not. Because if you tell people my story, there's a lot of key bullet points that would say that I shouldn't be successful (2, p. 22).

These bullet points are emblematic of a deficit narrative that all three youths described as a perpetual obstacle that impeded their social advancement, and that also impeded their mental health and wellbeing. Brian's conversing with the youth through the weekly afterschool program helped him to understand the multiple landscapes upon with these deficit narratives impacted youths. However, these youths also *retold* and *relived* stories according to their own personalized narratives that further exhibited their leadership, compassion, and resiliency.

## Shifting The Grand Narrative

The youths gradually felt comfortable sharing their stories with Brian once they had developed strong relationships through regular attendance at the weekly afterschool program. Brian noticed that the experiences lived and shared in the program were largely connected to feelings of belonging and fellowship that stood in contrast to the experiences of competition, winning, and losing that predominates many other youth sport programs. Instead of competition and aggression, the youths emphasized mentorship and role-modeling as key drivers of their involvement in the program, and as significant departures from the grand narrative associated with North Central Regina. For example, Clary explained how the afterschool program exhibited for youths an alternative narrative of education and career-development:

Yeah, I think it gives them something to look … like role model, like someone to look up to whether they're just doing a one-on-one situation or playing with them all. I think, sometimes, especially a lot of them that are First Nation kids, when I was a kid, you don't meet a lot of First Nation people that are successful in their high up careers like being a doctor and stuff like that. I think if they see high school students working toward going to university, and university students working toward their career goal, then they'll realize that that's okay and that's possible and whatnot. (1, p. 5)

*That's ok and that's possible* were words that resonated with the teachings of Joseph, an Elder affiliated with the afterschool program, who explained that “youths live the story they are told.” Clary's story centered on perseverance, resilience, and a desire to make a positive impression on the world. This was a story that was both shared and modeled by her mother—a role-model whom she tries to emulate through living her own mentorship story. As Clandinin (2013) argued, “as we retell or inquire into stories, we may begin to shift the institutional, social, and cultural narratives in which we are embedded” (p. 34). In this way, Clary re-lives the stories shared with her each time she works alongside other youths in the program, while simultaneously helping to rescript the grander narrative associated with North Central Regina.

### Bringing Belonging

The contrast between stereotypical portrayals of North Central Regina and the counter-narratives produced by Colin and the other youth mentors resurfaced again as both he and Brian strolled along the sidewalks near the schoolyard:

Yeah, we change the way we play for the kids who are playing with us, because some kids are not as skilled as the other kids. We always try to adapt ourselves to others so that we can also change the way our community is. We want a community that's good for everyone to grow up in, right? That's not the typical view of our community—it's considered a place that's bad for kids. Nothing is gonna change if we don't care for everyone (4, p. 14). Maybe it doesn't matter if you're the slowest one in the gym, you'll always be included. We all get a chance to be strong if we work together (3, p. 9).

For Colin, the lived and told stories of North Central Regina must be rewritten to promote inclusivity, not division. Colin redirected his energy and commitment to youth and community development to the afterschool program. For Colin, Candice, and Clary, the afterschool program afforded a prime opportunity to both express and impart the behaviors they valued, thereby contributing to rewriting the narrative associated with North Central. As Clary described:

I want the GYM program to instill a sense of belonging, because it's more like a family or sense of community. Because that's how community is, right? That's how family is, right? Everyone's not always the same age in a family. There's lots of differences and different things going on in a community, but we're still connected. That's how I want everyone to feel when they come to GYM, that they belong here. (4, p. 9)

Clary's reflection reminded Brian of the stories she had shared about her childhood. As noted above, Clary had a great deal of reverence for her mother, a single parent, who treated everybody equally and who supported the family through thick and thin. Clary modeled her relationship with others in the program in a similar fashion: “Supporting each other, that's what families do, right!” (Narrative account negotiation). Clary, along with the other mentors, not only shared this narrative with youth in the afterschool program, they lived it every day.

For example, Candice prided herself on being a “good” big sister who takes care of her siblings. She embraced a similar guardianship role in the afterschool program, and prided herself on making sure everyone felt safe and included in the various activities. Candice also explained how caring for others was not a burden, but a privilege that afforded her great joy:

I enjoy seeing kids have fun and play, so it kind of makes me feel happy. It makes me feel like if I play with them, it makes me feel young, I guess. I don't know. I like seeing little kids happy, so it makes me happy. (2, p. 12)

Candice further explained how she often felt lonely as a child, and identified the afterschool program as an opportunity to help prevent that feeling of loneliness in others: “It's important because we want all the kids to feel happy and feel like they belong. We make sure that everyone feels like they're being included in the activities. I know how it feels to not be included.” (3, p. 4) The undercurrent of Candice's ‘stories to live by' is rooted in a strong sense of guardianship, community, and a deep compassion for others. Her narrative prioritized nurturing a spirit of belonging among youths from diverse backgrounds and domestic situations—a teaching she credited to her Aunt, Ruth.

## Reimagining Teacher

As noted above, Candice dedicated herself to ensuring that all youths felt safe and valued in the afterschool program. Brian learnt that, from an early age, Candice had lived with her Aunt Ruth, who was in fact her foster parent. Affectionately known to all her foster children as “Auntie”, Aunt Ruth proved to be a tremendous role model and source of support for Candice and her foster siblings from the age of 8 years old. According to Candice, Ruth was caring and compassionate, and treated all her children equally. In addition, Ruth also taught Candice important life lessons about how to behave and act in public:

I think she taught me a lot about how to be respectful and an overall better person. She taught me how to be proper … like, teaching me how to have manners and that kind of thing. She also taught us all how to act in public, stuff like that. (1. p. 11)

Candice wanted to pass on Ruth's teachings to the other youths in the afterschool program, especially those who may not have enjoyed the same quality of mentorship that she had experienced as a youngster. For Candice, the afterschool program was about preparation for life beyond the gymnasium. In that sense, she prided herself on promoting teachings that went beyond physical activity within the afterschool program:

I want them to grow as individuals. That's important. Remember, we're also teaching them how to be polite, how to have manners, how to be respectful. School isn't really focusing on basic stuff like that, and that's kind of what we're doing so they learn those manners in the program and can better succeed in and outside of the school. (4, p. 9)

Of significance in the above excerpt is that Candice positioned herself as a teacher and as part of a larger teaching community accountable to those youths who showed up each week to the program. Candice also recognized Aunt Ruth as the source of her knowledge and she sought to honor her teachings through passing them on to others in the program.

Colin exhibited a similar commitment to teaching youths lessons that would help prepare them for life—lessons that he also believed were insufficiently addressed in school:

Well, in school, teachers usually teach what they think you should be learning. Education is what you should be learning, right? There's a difference. What they teach and what you will need in your future may be two entirely different things. Anyone can teach you math, like 1 + 1, but can math really teach you generosity? Those sorts of lessons are shown to you by family, friends, and community, right? Not in a classroom (1, p. 3)

For Colin, the lessons that were typically preached in school did not always square with the lessons required for life beyond the classroom environment. Education involved learning “real life stuff” (1, p. 2). “It's part of our life and begins the day we are born.” (1, p. 2). Like Candice, Colin credited a family member—Grandma Jean—with teaching him the most important lessons about love, compassion, and self-respect. “My grandma, she really helped me understand everything, like how to look out for others and treat people with respect. That's the *real* important stuff in life. That's the stuff that can take you places” (1, p. 10).

For both Candice and Colin, education unfolded across multiple landscapes; e.g., institutional, familial, and community landscapes. Colin shared an experience from his elementary school physical education class, which, incidentally, took place in the same gymnasium that hosts the afterschool program. Brian asked if the gymnasium felt any different from when Colin had attended the school as a youngster. Colin explained, “Well, there's only one teacher in a physical education class. But now, in our program, we've got five, six, seven teachers sometimes. Even the kids play a big role in teaching other kids. I think our program approaches teaching and learning as a community, not as an individual. That's a big difference. We also draw our teachings from everywhere, right? We're not reading a government curriculum. Our teachings come from us” (1, p. 16). When asked if he considered himself to be one of those teachers, Colin replied with a big smile across his face, “yeah, for sure.” He further explained,

A lot of these kids, they don't have older brothers. Or, if they do, they don't really interact with them. Everyone needs to look up to someone or something. When we're playing with these kids, we're helping them. By doing that, it puts us in the role of that older brother or older sister. I think that a lot of the younger kids look up to us older kids. They see us as teachers and Elders, or maybe as an older brother or sister. We have a responsibility to bring them along as best we can by sharing our best teachings. (1, p. 9)

Colin, Candice, and Clary all felt a great deal of pride and responsibility for mentoring youths in the afterschool program. They envisioned themselves as teachers, Elders, older siblings, and as part of a broader teaching and learning community invested in sharing their wisdom with the next generation of youths—an investment that they firmly believed would lead to positive social change both in and beyond their own lives.

## Concluding Remarks

In this article, we have tried to position Indigenous youths not as passive consumers of dominant messaging, but as co-authors of the afterschool program. In so doing, we have shed light on an area of youth development that remains underexplored; that is, how youths experienced an afterschool program intended to enhance their overall health and wellness (see Bruner et al., 2016). For Colin, Candice, and Clary, the program provided an opportunity to learn, teach, and grow as a community of youths invested in creating a better future. They saw themselves as teachers and knowledge holders, and they took great pride in sharing their life lessons with other youths, especially those in less fortunate circumstances. Finally, the lessons they drew upon were derived from a host of institutional, familial, and community landscapes.

As we have written about previously, (Schaefer et al., 2017), research partnerships such as the ones forged throughout this study require tremendous labor on behalf of all partners invested in the success of both the study and of the afterschool program. However, by including youths and community in the project from the outset, we observed an empowerment over the program that far exceeded our expectations and that proved instrumental to the program's ultimate success and salience in the community. Youths across all ages contributed to the vision and everyday operation of the program—helping to decide activities, organize outings, and contributing to quality and overall energy of their afterschool community. Consequently, the program is still running to this day in North Central Regina despite the termination of our initial funding. We sincerely hope this research affords important insights into the strength, resilience, and innovation of these youths who remain actively engaged in a struggle for personal, cultural, and community (re)construction against a backdrop of structural violence and urban marginality.

## Data Availability Statement

The original contributions presented in the study are included in the article/supplementary materials, further inquiries can be directed to the corresponding author/s.

## Ethics Statement

The studies involving human participants were reviewed and approved by University of Regina, REB. Written informed consent to participate in this study was provided by the participants' legal guardian/next of kin.

## Author Contributions

LS was BL's Ph.D. supervisor while this research was conducted. SL contributed to this work, as well as earlier drafts of this paper. JK was added as an author at a later date, and contributed quite significantly to this final draft. All authors contributed to the article and approved the submitted version.

## Conflict of Interest

The authors declare that the research was conducted in the absence of any commercial or financial relationships that could be construed as a potential conflict of interest.

## Publisher's Note

All claims expressed in this article are solely those of the authors and do not necessarily represent those of their affiliated organizations, or those of the publisher, the editors and the reviewers. Any product that may be evaluated in this article, or claim that may be made by its manufacturer, is not guaranteed or endorsed by the publisher.

## Footnotes

^1^This program is still ongoing.

^2^Candice, Clary, and Colin are negotiated pseudonym names of the research participants.

^3^Format used for referencing transcribed research conversations (Conversation 3, page 19).
